# Sublingual Immunotherapy: Clinical Indications in the WAO-SLIT Position Paper

**DOI:** 10.1097/WOX.0b013e3181e8d19c

**Published:** 2010-07-15

**Authors:** Giovanni Passalacqua, Enrico Compalati, Giorgio Walter Canonica

**Affiliations:** 1 Allergy and Respiratory Diseases, DIMI, University of Genoa, Genoa, Italy

**Keywords:** sublingual immunotherapy, SLIT, allergic rhinitis, clinical indications for SLIT, SLIT methodology for clinical trials

## Abstract

Sublingual immunotherapy (SLIT) is a matter of only 20 years. Nonetheless, in this short period of time more than 60 randomized double blind placebo-controlled trials have been published, in addition to postmarketing surveillance studies and meta-analyses. The wide diffusion of SLIT in clinical practice and the large availability of experimental data prompted the WAO to publish a position paper on SLIT, to identify the indications, contraindications, and practical aspects of the treatment. On the basis of the available literature, SLIT is certainly indicated in allergic rhinitis in both adults and children. In this latter population, SLIT may exert a preventative effect on the development of asthma. The age seems not to represent a special problem. SLIT can be used also when asthma is associated to rhinitis, whereas it is not the first choice for the treatment of isolated asthma. The IgE-mediated mechanism and the clear identification of the causal role of the allergen are mandatory prerequisites for prescribing SLIT. The safety profile is excellent, but it is recommended that the first dose be given under medical supervision. Atopic dermatitis, latex allergy, and hymenoptera hypersensitivity are promising fields of use of SLIT, but they are still considered only experimental uses.

## Introduction

The subcutaneous modality of immunotherapy injections (SCIT) remained, for several decades, as the only available administration route, although it is burdened by a certain risk of severe side effects. The problem of the risk/benefit ratio prompted the search for safer administration routes including the sublingual one, sublingual immunotherapy (SLIT), which was first described in 1986 [1]. In less than 20 years, a very large amount of clinical data, controlled trials, and postmarketing surveys on SLIT were published. As such, SLIT progressively achieved credibility and was introduced in the official documents as a viable alternative to the classic injection route [2,3] for both adults and children. Because of the increasing utilization of SLIT in Europe and many other countries worldwide, it was felt that a document or position paper on its clinical use, safety aspect, and unmet needs was necessary. For this reason, the World Allergy Organization convened a meeting in Paris during January 2009 to prepare and discuss a position paper. This was released as a draft within June 2009 and subsequently endorsed by almost all the national allergy societies and all the large regional societies. The WAO-SLIT position paper was presented in the *WAO Journal*[4] just before the 2009 World Allergy Congress in Buenos Aires and published as a supplement in *Allergy *in December 2009 [5].

The document reviews the currently available literature on SLIT, including efficacy, safety, mechanisms, impact on the natural history, indications, methodology for clinical trials, and unmet needs. From the clinician's viewpoint, the indications to SLIT and the choice of eligible patients is of primary relevance. The indications suggested in the position paper are derived as far as possible from the experimental evidence available.

### Clinical Efficacy and Safety: The Literature

At the date of publication of the position paper there were 60 randomized double blind placebo-controlled trials performed with SLIT and various meta-analyses. The meta-analyses included patients with rhinitis only,[6] asthma only,[7] and asthma and rhinitis in children [8,9]. All the meta-analyses concluded that there was a significant effect of SLIT versus placebo. The reliability of the meta-analyses has been questioned[10] especially on the basis of possible publication biases, incorrect reporting of the data, and large heterogeneity of the trials included. The problem of heterogeneity has been repeatedly highlighted as a drawback, but it is also true that meta-analyses are intended to summarize the results of studies when they are not directly comparable to each other. Furthermore, the mentioned meta-analyses pooled together the studies with all allergenic extracts, whereas differences may exist among allergens. In this regard, there is now a meta-analysis restricted to house dust mite SLIT that shows a significant effect on symptoms [11].

The so-called "big trials" (for review[4,5]), conducted with grass pollen extracts, provided relevant information on the clinical use of SLIT. In particular, the clinical effect of SLIT versus placebo was shown to range between 25% and 50% when the cutoff for efficacy is set at 20% [12]. More importantly, the big trials demonstrated that the clinical effect is to some extent dose-dependent and that the optimal maintenance dose for grass SLIT is around 30 times the corresponding dose used with the injection route.

The effect in asthma is still a matter of debate because some trials[13-15] reported a marginal or no effect on asthma symptoms. It is also true that the Dahl study[13] was designed to assess the safety, and in the Pham-Thi trial[14] all the patients (active and placebo) had no symptoms of asthma at baseline nor during the trial and therefore no effect could be seen. When patients with current asthma symptoms are studied, the effect of SLIT can be detected. An early study[16] in adults and adolescents reported an improvement in quality of life and respiratory function and a decrease in inhaled steroid use. Three pediatric studies[17-19] with mite SLIT found a significant difference between active and placebo patients in day-time and nighttime symptoms, and 1 study also demonstrated a reduction in the eosinophil count [18]. Those results were recently replicated in another pediatric trial with grass SLIT [20]. Finally, in some studies, SLIT was shown capable of reducing the degree of bronchial hyperresponsiveness [21,22].

SLIT, similarly to SCIT, can prevent the onset of new sensitizations,[23] and also prevent the onset of asthma in children with rhinitis [24,25]. In addition, there are also 3 studies that have demonstrated a long-lasting effect of SLIT after discontinuation [26-28]. Of note, the study by Durham et al[28] deals with the follow-up at 1 year of the patients included in one of the big trials.

Concerning the other possible indications of SLIT, there are some controlled trials reporting a satisfactory efficacy profile in latex allergy[29,30] and in food allergy,[31,32] whereas there is 1 single randomized controlled trial of SLIT with house dust mite in children with atopic dermatitis [33]. The possible use of SLIT has been envisaged also in hymenoptera venom allergy,[34] but also in this case there is 1 single controlled trial dealing with large local reactions.

The safety of SLIT is superior to that of SCIT,[35] and no fatality has been reported in 23 years of trials and clinical use. The most frequently reported events were irritation of the throat and oral itching. According to the recent data, the number of side effects seems to be dose-dependent, as happens with SCIT. There are only *6 *reports of anaphylaxis with SLIT, although the occurrence of anaphylaxis at the first dose would suggest the opportunity of giving the first dose under medical supervision. In a recent report,[36] 2 severe reactions (not anaphylactic shock) were described in 2 patients with previous severe reactions to SCIT. Thus, caution should be taken in patients with previous adverse events who are switched to SLIT. Finally, some postmarketing surveys confirmed that the safety of SLIT does not change in children younger than 5 years [37,38].

### Indications (Table 1)

**Table 1:** Criteria of Selection for SLIT (Adapted From[4,5])

• To be eligible for SLIT, patients should have the follow:
A clinical history of allergy.
Documented ALLERGEN-SPECIFIC IgE positive test.
The allergen used for immunotherapy must be clinically relevant to their clinical history.
• Age does not seem to be a limitation.
• Monosensitized patients are ideal candidates for SLIT, and recently single-allergen SLIT has been demonstrated to be effective in polysensitized patients.
• Presently, use of SLIT in latex allergy, atopic dermatitis, food allergy, and hymenoptera venom allergy is under investigation: more demonstrations are needed to support clinical use.
• There is no indication whatsoever for treating non-IgE-mediated hypersensitivity (i.e., nickel sensitivity) with SLIT.
• SLIT may be considered as initial treatment; failure of pharmacological treatment is not an essential prerequisite for the use of SLIT.
• SLIT may be proposed as an early treatment in respiratory allergy therapeutic strategy.
• Special SLIT indications exist in the following patients:
Patients uncontrolled with optimal pharmacotherapy (SCUAD).
Patients in whom pharmacotherapy induces undesirable side effects.
Patients refusing injections.
Patients who do not want to be on constant or long-term pharmacotherapy.

It is mandatory that the IgE-mediated mechanism of the disease (rhinitis or asthma) be clearly established, in addition to the causal relationship between the exposure to allergen and symptoms. This remains true for subcutaneous immunotherapy and for SLIT.

On the basis of the experimental evidence summarized above, the indication to SLIT in allergic rhinitis is well established in adults and children, independent of the allergen considered. This also takes into account the very favorable safety profile of SLIT. In addition, it can potentially modify the disease and the clinical benefits may be sustained years after discontinuation of treatment. Concerning asthma, SLIT should be considered if symptoms are persistent, *despite pharmacological and nonpharmacological measures, or when medications cause unacceptable side effects or patients refuse to use inhaled corticosteroid*. Also in this case immunotherapy should be considered only if there is clear evidence of a relationship between symptoms and exposure to an allergen to which the patient is sensitive. *Similarly to SCIT, also with SLIT severe/uncontrolled asthma is a contraindication*. Patients allergic to mites may be candidates for SLIT if they have significant symptoms of rhinitis or asthma when they are exposed to domestic mite allergens.

Concerning children, SLIT is effective in allergic rhinitis in children older than 5 years and may be safe in allergic rhinitis in children older than 3 years. Thus, SLIT can be used for allergic rhinitis in children with or without asthma but should not be suggested as monotherapy for treating asthma.

Presently the use of SLIT in latex allergy, atopic dermatitis, food allergy, and hymenoptera venom allergy is under investigation, and more demonstrations are needed to support the clinical use.

### Unmet Needs

Some aspects of SLIT need to be elucidated, to provide clinicians with clear and evidence-based recommendations for the clinical use of the treatment. The most compelling problem is the variability of the doses used in clinical trials because both positive and negative results have been obtained with both low and high doses and the dose interval for efficacy is reported to range between 2 and 375 times the amount given with SCIT. This is complicated by the variability in the protein content of the extracts by different manufacturers [39]. Only for grasses has an optimal dose been identified in 15-25 *µ*g of major allergen per day, which is roughly 50 times the monthly dose of SCIT. Dose-response trials and the identification of the optimal maintenance dose are needed at least for the more relevant allergens. From a clinical point of view, there is no consensus on which is the best administration regimen among the pre-seasonal, co-seasonal, pre-co-seasonal, or continuous. It is true that, for pollen allergens, most of the trials used a pre-co-seasonal regimen,[40] but this cannot be immediately extrapolated to all extracts and to all patients. Similarly, the usefulness of a build-up phase is still a matter of debate.

Finally, it is mandatory that all future trials be properly designed, with a sample size calculation and with clearly established outcomes,[41,42] permitting better standardization of treatment and providing clinicians with more univocal guide-lines in the near future.
